# Importance of underweight in childhood bacterial meningitis in Finland, Latin America and Angola

**DOI:** 10.1038/s41598-022-15131-8

**Published:** 2022-06-29

**Authors:** Irmeli Roine, Markku Kallio, Heikki Peltola, Tuula Pelkonen

**Affiliations:** 1 Faculty of Medicine, University Diego Portales, Santiago, Chile; 2 University of Helsinki, and Children’s Hospital, Helsinki University Central Hospital, Helsinki, Finland; 3 New Children’s Hospital, Pediatric Research Center, University of Helsinki and Helsinki University Hospital, P.O. Box 347, 00029 HUS Helsinki, Finland; 4 Hospital Pediátrico David Bernardino, Luanda, Angola

**Keywords:** Diseases, Infectious diseases, Bacterial infection, Meningitis, Nutrition, Paediatrics

## Abstract

Our objective was to explore the importance of underweight on the course of childhood bacterial meningitis (BM) at different study sites, because prior studies showed discrepant results. Using directly comparable, prospective data from three continents, weight-for-age z-scores (WAZ) were determined by WHO Anthro programs in children with BM in Finland (N = 318), LatAm (N = 580), and Angola (N = 780) and compared with data describing the admission, course, and outcome of BM. WAZ < –1 indicates underweight; either mild (< –1 to –2), moderate (< –2 to –3), or severe (< –3). The mean WAZ (SD) was 0.17 (1.17), –0.42 (1.53), and –1.36 (1.44), and the prevalence of moderate-severe underweight 2.8%, 12.6%, and 31.3%, in Finland, LatAm, and Angola, respectively. In univariate analysis, LatAm and Angola showed an association between lower WAZ and poorer condition on admission, slower recovery, and more deaths. In Finland, infrequent underweight limited meaningful analysis. In multivariate analysis of different variables for increasing the odds of death, severe underweight had lower odds compared to disease severity in Angola, but highest in LatAm. Thus, the apparent discrepancy in underweights´ importance for increasing deaths varied from primary to more secondary according to locally more prominent risks.

## Introduction

Bacterial meningitis (BM) is the 10th most common cause of mortality in children under 5 years of age^1^ despite effective vaccines against the three major agents. The incidence, mortality, and sequelae rates from BM are highest in the areas with slowest socio-economic development^2–7^. Mortality relative to Finland has been fourfold in Latin America (LatAm) and 11-fold in Angola^3–7^.

Apart from in-hospital treatment and etiology, the outcome of childhood BM is strongly influenced by conditions on presentation such as delay in getting to hospital^8^, prior seizures^8^, arrival with a low Glasgow Coma Score^9^, and a low weight-for-age^10^. Interventions aimed at improving the outcome of BM by dealing with these components may hold more promise than, say, new treatments. Regarding underweight, it is unclear whether it´s prognostically negative impact can be diminished by some management in the hospital.

Undernutrition debilitates both the innate and the adaptive immune response and increases mortality in general, but especially of infectious diseases^11^. In previous studies of “malnutrition” in BM, its impact in increasing deaths has varied. In LatAm, mortality was increased already by mild underweight in under 5-year-olds^10^, whereas in Angola not even clinically determined severe underweight increased deaths in a retrospective analysis^8^. We questioned whether the difference derived from data that were not directly comparable or, hypothetically, underweight could produce dissimilar effects in different populations. To re-examine and compare the role of underweight, we used prospectively collected, comparable data from BM on three continents. Here we describe the prevalence and associations of underweight with the course of BM comparing a group of BM patients in Finland, LatAm, and Angola.

## Methods

### Study design and ethical aspects

This is a secondary, descriptive analysis of prospectively collected data from five clinical treatment trials carried out on three continents in 1984–2017 in an attempt to improve the outcome of childhood BM^3–7^. The Luanda Children’s Hospital’s Ethics Committee approved the studies in Angola, and the relevant Ethics Committees or Hospital Boards approved the studies in 6 countries in Latin America and in 12 hospitals in Finland. Once the registration of clinical trials commenced, the last two were registered (ISRCTN62824827 and NCT01540838). The patients were enrolled after written or oral informed consent was obtained from the guardian. All methods were carried out in accordance with the Declaration of Helsinki.

### Study settings and participants

Details of the studies have been reported earlier^3–7^. In brief, all patients aged 2 months to 15 years with probable BM were consecutively enrolled, treatments started, and the admission and follow-up data registered by attending physicians. The treatment allocations were blinded and at random. The diagnosis of BM was considered confirmed if (1) cerebrospinal fluid (CSF) culture or PCR proved positive for a bacterial cause of meningitis, (2) the patient showed compatible symptoms and signs and a bacteria identified by blood culture, Latex, or Gram result, or (3) in addition to compatible symptoms and signs (tense fontanelle/neck rigidity, impaired consciousness, absent look, irritability, vomiting), two laboratory criteria, as detailed in each study´s publication (cerebrospinal fluid [CSF] pleocytosis > 1000/ mm^3^, CSF glucose level < 40 mg/l, CSF protein level ≥ 40 mg/l, blood C-reactive protein > 40 mg/l, or blood leucocyte count > 15,000/mm^3^), supported the diagnosis^3–7^. None of the tested treatment alternatives diminished deaths, although severe neurological sequelae were significantly reduced by oral glycerol in Latin America (Odds Ratio [OR] 0.31, 95% confidence interval [95% CI] 0.13–0.76)^5^. The underweight patients had been equally distributed in the different treatment arms in each of the original studies [6,7, and in 3–5 as checked for the present publication].

The present study included all patients with confirmed BM aged from 2 months to 10 years with a recorded date of birth or an exact age (Table 1). The previous report from LatAm^10^ was limited to patients with a recorded date of birth under 5 years of age, which was the upper limit of age in the then only available WHO Anthro program^12^.

**Table 1:** Patient characteristics.

	Finland	Latin America	Angola	*p* value
Confirmed bacterial meningitis, all	N = 351	N = 654	N = 822	
**Reason for exclusion**				
Age above 120 months	23 (7)^a^	50 (8)	33 (6)	
Admission weight not registered	10 (3)	24 (4)	9 (1)	
Included in analysis	318 (91)	580 (89)	780 (95)	< 0.0001
Weight-for-age by exact dates	318 (100)	545 (94)	773 (99)	
Weight-for-age by age in months	··	35 (6)	7 (1)	
Age in years, median	1.8 [0.8–3.3]^b^	0.8 [0.4–2.2]	1.0 [0.5–2.9]	< 0.0001
Females	148/318 (47)	246/580 (42)	356/780 (46)	0.38
**Weight-for-age z-score, mean ± SD**	0.17 ± 1.17	− 0.42 ± 1.53	− 1.36 ± 1.44	< 0.0001
Normal weight for age (64.2)^c^	213 (67.0)	312 (53.8)	283 (36.3)	
Mild underweight (13.6)^c^	32 (10.1)	111 (19.2)	213 (27.3)	
Moderate underweight (2**.**1)^c^	5 (1.6)	41 (7.1)	143 (18.3)	
Severe underweight (0.1)^c^	4 (1.2)	32 (5.5)	101 (13.0)	
Simple overweight (13.6)^c^	48 (15.1)	54 (9.3)	36 (4.6)	
Obesity (2.1)^c^	12 (3.8)	24 (4.1)	3 (0.4)	
Severe obesity (0.1)^c^	4 (1.2)	6 (1.0)	1 (0.1)	
Death	9/318 (3)	79/580 (14)	294/780 (38)	< 0.0001
Severe neurological sequelae^d^	4/309 (1)	42/486 (9)	67/483 (14)	< 0.0001
Deafness^e^	0/255 (0)	48/400 (12)	47/436 (11)	< 0.0001
Any neurological sequelae^f^	56/309 (18)	145/484 (30)	220/477 (46)	< 0.0001
Any hearing impairment^g^	19/255 (7)	144/400 (36)	150/296 (51)	< 0.0001

^a^In parentheses, percentage. ^b^Interquartile range. ^c^Expected proportion in a normal distribution of z-scores of weight-for-age. ^d^Blindness, quadriplegia/paresis, hydrocephalus requiring a shunt, or severe psychomotor retardation. ^e^Hearing threshold in the better ear ≥ 80 dB. ^f^Severe neurological sequelae, or moderate psychomotor retardation, hemiparesis, monoparesis, or ataxia. ^g^Hearing threshold > 40 dB in either ear.

### Data collection

The data from the five trials were prospectively collected on similar forms, the predefined definitions describing the clinical course and outcome were the same, and all were overseen by the same person (H.P.), allowing direct comparisons between the studies.

At discharge, the clinical course was graded as ordinary, complicated, or fatal (see Fig. 2). Neurological sequelae were evaluated by the attending physician. Both ears’ hearing was measured by brain evoked response audiometry or audiometry, the child´s age permitting. The findings were categorized (definitions in Table 1) as severe or any neurological sequelae, deafness or any hearing loss, and death.

Body weight was registered on admission. Using the date of birth, or exact age in months, the weight-for-age z-score (WAZ-score, a standard deviation score) was determined by the WHO international growth standard calculators Anthro and AnthroPlus^11,12^. The growth data in these programs stem from a study of internationally diverse populations of children whose care needs were met and whose growth reflected the current health recommendations (“optimal growth”)^14^. In Angola, the attending physician registered also his or her clinical impression of probable “malnutrition”, distinguishing it as any, moderate, or severe.

**Figure 1:**
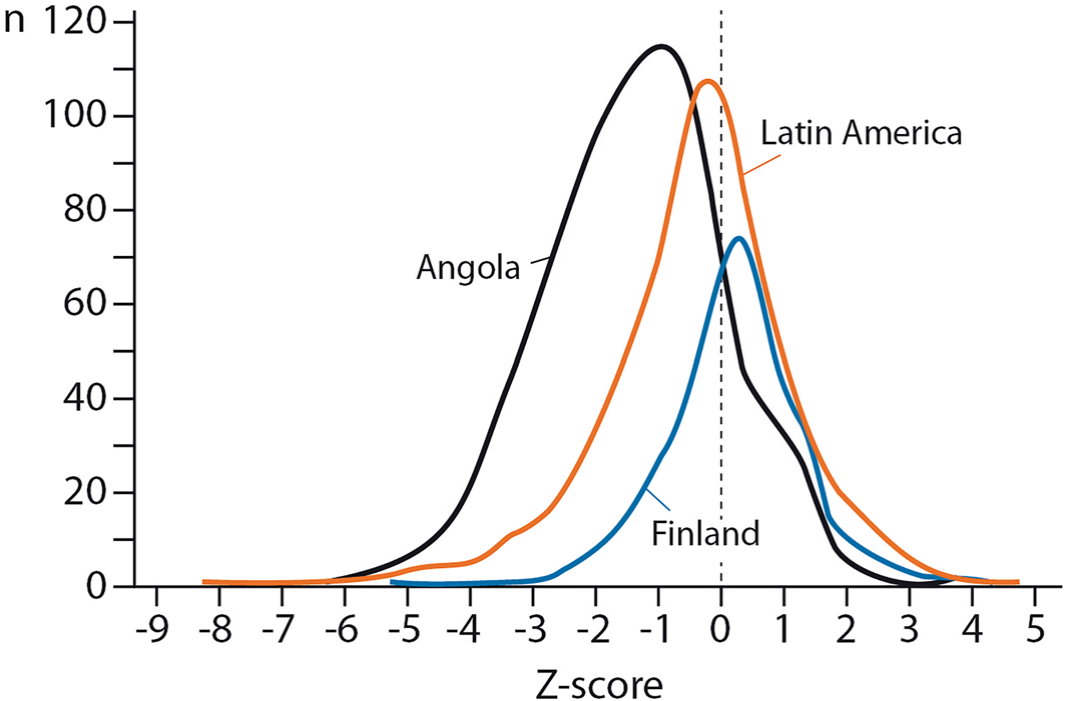
Distribution of weight-for-age z-scores in Finland, Latin America, and Angola.

A complete nutritional and growth evaluation requires, in addition to weight, measurements of length/height and skin fold thicknesses, which were not available for us. A WAZ result by itself expresses the extent to which the patient´s weight differs from the mean of his/her age, as expressed in standard deviations from the mean. Importantly, it does not identify a more specific cause of growth failure, such as acute or chronic undernutrition. The WAZ-scores during optimal growth show a normal distribution with known proportions of the results in and beyond the mean ± one standard deviation in the center and on both sides, as in any curve with a normal distribution (Table 1, Fig. 1)^15^. In Anthro and AnthroPlus, a WAZ-score < − 1 is considered underweight, which is subdivided into the categories of mild (WAZ-score < − 1 to − 2), moderate (WAZ-score < − 2 to − 3), and severe (WAZ-score < − 3)^12,13^. Likewise, a WAZ-score >  + 1 is considered overweight, subdivided into simple overweight (> + 1 to + 2), obesity (> + 2 to + 3), and severe obesity (> + 3).

We compared the prevalence of the different degrees of underweight in our patients with those found in optimal growth according to WHO^14^. A similar prevalence would show that there were no more children with a low weight-for-age than expected in a normal distribution of healthy children of different sizes. Clearly higher figures, by contrast, indicate undernutrition. To examine the potential role of weight-for-age on the course of BM, we compared the WAZ-scores with other patient findings on admission, during hospital stay, and for outcome.

### Statistical analysis

Continuous variables were expressed as means with SD or medians with interquartile range (IQR), as appropriate, and qualitative variables with numbers and percentages. Comparisons between WAZ-scores and other variables describing the findings on admission and during the course of BM were carried out using Student´s t-test, ANOVA, Spearman’s correlation, or contingency table, as appropriate. The OR for death were calculated by the JMP ® Pro 14.1.0 (SAS Institute Inc, Cary, NC, USA) for Windows program with 95% CI using the WAZ-score categories as an independent variable in univariate analysis, and in multivariate analysis, together with other, most relevant independent predictors according to our previous analysis from the same data (treatment delay > 3 days, prior seizures, Glasgow Coma Score < 13, and WAZ < -3)^8–10^. Tests and CIs on ORs are Wald-based. Taking into account multiple testing, a *p* < 0.01 was considered as significant and a *p* < 0.05 but ≥ 0.01 to indicate a trend towards significance.

### Ethics approval and consent to participate

The Luanda Children’s Hospital’s Ethics Committee approved the studies in Angola, and the relevant Ethics Committees or Hospital Boards approved the studies in 6 countries in Latin America and in 12 hospitals in Finland. Once the registration of clinical trials commenced, the last two were registered (ISRCTN62824827 and NCT01540838). The patients were enrolled after written or oral informed consent was obtained from the guardian.

## Results

Of the patients in the original studies (Table 1), 1678 (92%) fulfilled the inclusion criteria for the present analysis. Causes for exclusion were age over 10 years (*n* = 106) and lack of the required data for the WAZ-score determination (*n* = 43). The WAZ-scores were determined according to the exact date of birth in 97.5% and by age in 2.5% of cases. The diagnosis of BM was confirmed by finding bacteria with blood or CSF culture, PCR, Latex or Gram stain in 100%, 86%, and 79% of patients in Finland, LatAm and Angola, respectively.

Patients on the three study sites did not differ in sex distribution (*p* < 0.05), and although there was a difference in age, the medians were similar, with slightly older patients in Finland (Table 1).

### WAZ-score results and prevalence of different stages of undernutrition according to study site

The WAZ-scores had a normal distribution on all study sites but their mean values (SD) were clearly (Fig. 1, *p* < 0.0001) different: 0.17 (1.17), − 0.42 (1.53), and − 1.36 (1.44) in Finland, Latin America, and Angola, respectively.

In optimal growth (Table 1)^12,13^, 15.8% of children are smaller than mean  1 SD and therefore expected to have a WAZ-score < − 1. The equivalent figures in our patients were 12.9% in Finland, but 31.8% in LatAm, and 58.6% in Angola. Likewise, in optimal growth, a WAZ-score < − 2 is an expected finding in the smallest 2.2% of children. In comparison, the equivalent figures were 2.8% in Finland, but 12.6% in LatAm and 31.3% in Angola. Compared with Finland, LatAm patients were 4.10 (95% CI 2.09–8.07) times more likely to have a WAZ < − 2, indicating moderate-severe underweight^12,13^. In Angola, the corresponding odds were 12.81 (95% CI 6.73–24.38). In Angola (not registered in Finland or LatAm), the clinician´s impression of “any malnutrition” corresponded to a WAZ-score of − 2.53 (SD 1.39), indicating at least moderate undernutrition. The patients whose clinical impression was “severe malnutrition” had a WAZ-score of − 3.62 (SD 1.24).

### WAZ-score associations with other variables according to study site

At all three study sites, a lower WAZ-score (Table 2) associated with a lower systolic blood pressure on admission, and corresponded to more of any neurological sequelae at discharge (*p* = 0.005, 0.006, 0.046 and *p* = 0.019, 0.025, 0.038 respectively, for blood pressure and any neurological sequelae, in Finland, LatAm and Angola). Although the age of the patients also correlated significantly with the WAZ-score, in Finland and LatAm higher age correlated with a higher WAZ-score, but in Angola with a lower WAZ-score.

**Table 2:** Associations between weight-for-age z-score and other findings in children with bacterial meningitis from Finland, Latin America, and Angola.

	Finland	Latin America	Angola	*p* value
	Rho^a^ or *p* value	Rho or *p* value	Rho or *p* value	
	z-score^b^	z-score	z-score	
Age in months	0.15 0.007	0.12 0.003	− 0.14 < 0.0001	0.95
Female sex *vs.* male sex	Lower 0.75	Higher 0.004	Higher < 0.0001	< 0.0001
**Before admission**				
Days ill	− 0.01 0.83	− 0.13 0.020	− 0.10 0.004	0.0003
Antimicrobials *vs.* no	Higher 0.058	Lower 0.64	Lower 0.11	0.41
Convulsions *vs.* no	Higher 0.72	Lower 0.009	Lower 0.57	0.27
**On admission**				
Underlying condition *vs.* no	Lower 0.001	Lower 0.59	Lower 0.003	< 0.0001
Poor general condition *vs.* no	Lower 0.80	Lower < 0.0001	Lower 0.049	< 0.0001
Glasgow Coma Score	NA	0.18 < 0.0001	0.05 0.20	0.0003
Capillary filling time	NA	− 0.12 0.009	− 0.19 0.014	0.013
Systolic blood pressure	0.18 0.005	0.13 0.006	0.07 0.047	0.0001
Heart rate	− 0.12 0.039	− 0.06 0.14	0.02 0.64	0.15
Other focus of infection *vs.* no	Lower 0.17	Lower 0.56	Lower 0.004	0.001
CSF^c^ WCC^d^	0.009 0.88	− 0.04 0.39	− 0.02 0.67	0.23
“ Glucose	0.11 0·047	0·10 0·020	0.07 0.07	0.099
“ Protein	0.04 0.51	− 0.10 0.034	− 0.08 0.26	0.053
Blood haemoglobin	0.17 0.003	0.12 0.005	0.07 0.06	< 0.0001
Blood WCC^d^	− 0.09 0.88	0.03 0.51	− 0.08 0.15	0.71
Blood glucose	NA	0.03 0.46	0.06 0.12	0.42
**In hospital**				
Days of fever > 37.5 °C	0.01 0.86	− 0.03 0.53	0.02 0.61	0.50
Aetiology	0.081^e^	0.020	0.49	0.51
*S. pneumoniae*	Intermediate	Lowest	Highest	
*H. influenzae*	Highest	Intermediate	Lowest	
*N. meningitidis*	Lowest	Highest	Intermediate	
GCS^f^ < 15, days	− 0.03 0.60	− 0.14 0.002	− 0.09 0.012	0.037
Days of stay	− 0.06 0.27	− 0.04 0.51	− 0.03 0.44	0.16
**Outcome at discharge**				
Glasgow Outcome Score	NA	0.25 < 0.0001	0.08 0.036	< 00001
Death *vs.* survival	Lower 0.70	Lower < 0.0001	Lower 0.11	< 0.0001
Severe neurological sequelae	Lower 0.032	Lower 0.073	Lower 0.71	0.035
Deafness	NA	Higher 0.88	Lower 0.88	0.86
Any neurological sequelae	Lower 0.019	Lower 0.025	Lower 0.038	< 0.0001
Any hearing impairment	Lower 0.076	Higher 0.95	Lower 0.65	0.84

^a^By Spearman correlation. ^b^Whether the z-score mean value in patients with the indicated variable was higher or lower *vs.* patients without it, as analyzed by Student´s t-test., ^c^Cerebrospinal fluid. ^d^White cell count. ^e^by ANOVA. ^f^Glasgow Coma Score.

In LatAm and Angola, several of the same variables showed significant associations with the WAZ-scores, albeit with somewhat higher rho values (= correlation coefficients in non-parametric regression) and smaller *p* values in Latin America. In both, females showed higher WAZ-scores (*p* = 0.004 and *p* < 0.0001, in LatAm and Angola, respectively), whereas lower WAZ-scores characterized patients who arrived at hospital in a poorer general condition (*p* < 0.0001 and *p* = 0.049, in LatAm and Angola, respectively), had been ill for more days before admission (*p* = 0.02 and *p* = 0.04, in LatAm and Angola, respectively), and showed a longer capillary filling time (*p* = 0.009 and *p* = 0.014, in LatAm and Angola, respectively). Lower WAZ-scores also portrayed patients who’s in-hospital recovery was slower (*p* = 0.002 and *p* = 0.012, in LatAm and Angola, respectively), as indicated by more days with a Glasgow Coma Score (3–15, best) < 15. The Glasgow Outcome Score (1–5, best) was higher in patients with higher WAZ-scores in both Latin America and Angola (*p* < 0.0001 for both).

In Finland and LatAm, blood hemoglobin (*p* = 0.003 and *p* = 0.005, respectively) and CSF-glucose (*p* = 0.047 and *p* = 0.02, respectively) concentrations were lower in patients with lower WAZ-scores but not in Angola. In LatAm (Table 2), but not in Angola (not determined in Finland), a lower admission Glasgow Coma Score was associated significantly with a lower WAZ-score (*p* < 0.0001). Patients with pneumococcal meningitis (*p* < 0.0001) and prior convulsions (*p* = 0.009) had lower WAZ-scores than patients without them. Further, in LatAm a lower CSF-protein level correlated with a higher WAZ-score (*p* = 0.03) and patients who died *versus* survivors had lower WAZ-scores (*p* < 0.0001).

HIV antibody (only determined in Angola) positivity was associated with a significantly lower WAZ-score (*p* = 0.006). Excluding the HIV positive patients from the above analyses did not change the results (data not shown). Deafness or any hearing sequelae were not associated (*p* > 0.05) with the WAZ-score.

In a univariate analysis comparing death with the different stages of underweight (Table 3), mild underweight increased deaths 2.09 (95% CI 1.15–3.80) times and severe underweight 5.31 (95% CI 2.38–11.85) times in Latin America. In Angola, only severe underweight increased deaths 1.92 (95% CI 1.21–3.04) times. In Finland, the paucity of data restricted the relevance of the results. In all patients combined, the odds for death increased from mild to moderate to severe underweight by ratios of 1.52 (95% CI 1.13- 2.06), 1.97 (95% CI 1.38–2.83), and 4.38 (95% CI 3.09–6.41), respectively. Although severe obesity did not increase mortality significantly at any study site, in LatAm it showed a possible trend towards this with an odds ratio of 4.32 (95% CI 0.78–25.10).

**Table 3:** Odds of the different degrees of under- or overweight, compared with children with normal weight-for-age, in relation to death in children with bacterial meningitis in Finland, Latin America, and Angola.

	Finland	Latin America	Angola	All
	OR^*a*^* 95%* CI^*b*^	OR 95% CI	OR 95% CI	OR 95% CI
Mild underweight	··	2.09 1.15–3.80	0.78 0.54–1.14	1.52 1.13–2.06
Moderate underweight	··	2.08 0.89–4.88	0.90 0.59–1.37	1.97 1.38–2.83
Severe underweight	9.86 0.91–107.06^c^	5.31 2.38–11.85	1.92 1.21–3.04	4.38 3.00–6.41
Simple overweight	0.62 0.08–5.21	0.52 0.15–1.76	0.94 0.46–1.94	0.64 0.37–1.10
Obesity	··	0.80 0.18.3·58	3.34 0.30–37.28	0.52 0.18–1.49
Severe obesity	··	4.42 0.78–25.10	··	1.71 0.45–6.54

^a^Odds ratio. ^b^Confidence intervals. ^c^One of four patients in this category died.

### Risk of death in multivariate analysis according to study site

In a multivariate analysis (Table 4) exploring the relative importance of different factors for increasing deaths, the independent variables were a Glasgow Coma Score < 13, treatment delay > 3 days, prior seizures, and severe underweight^8–10^. The most prominent predictor in LatAm was severe underweight (OR 6.69, 95% CI 1.88–23.86), in contrast to Angola, where Glasgow Coma Score < 13 (OR 5.68, 95% CI 3.35–9.61) was most important. Again, in Finland the paucity of both underweight and deaths limited the relevance of the results.

**Table 4:** Multivariate analysis of variables associated with death from childhood bacterial meningitis in Latin America and Angola.

	Latin America	Angola	All
	OR^a^ 95% CI^b^	OR 95% CI	OR 95% CI
Glasgow Coma Score < 13 *vs.* ≥ 13	3.95 1·23–12.65	5.68 3.35–9.61	4.74 3.07–7.31
Treatment delay > 3 days *vs.* ≤ 3 days	1.89 0.65–5.54	1.20 0.70–1.94	2.09 1.41–3.08
Prior seizures *vs.* no	4.20 1.35–13.09	1.31 0.80–2.15	2.38 1.60–3.55
Weight-for-age z-score < − 3 *vs*. + 1 to − 1	6.69 1.88–23.86	2.11 1.25–3.56	4.05 2.52–6.51

A comparison of the influence of severity on admission (Glasgow Coma Score ˂ 13), treatment delay > 3 days, seizures before or during admission, and severe underweight (= weight-for-age z-score < − 3) *vs*. normal weight (weight-for-age z-score + 1 to − 1). In Finland, too few deaths impeded multivariate analysis. ^a^Odds ratio. ^b^Confidence intervals.

### Associations between different stages of underweight with other variables, in all patients

**Figure 2:**
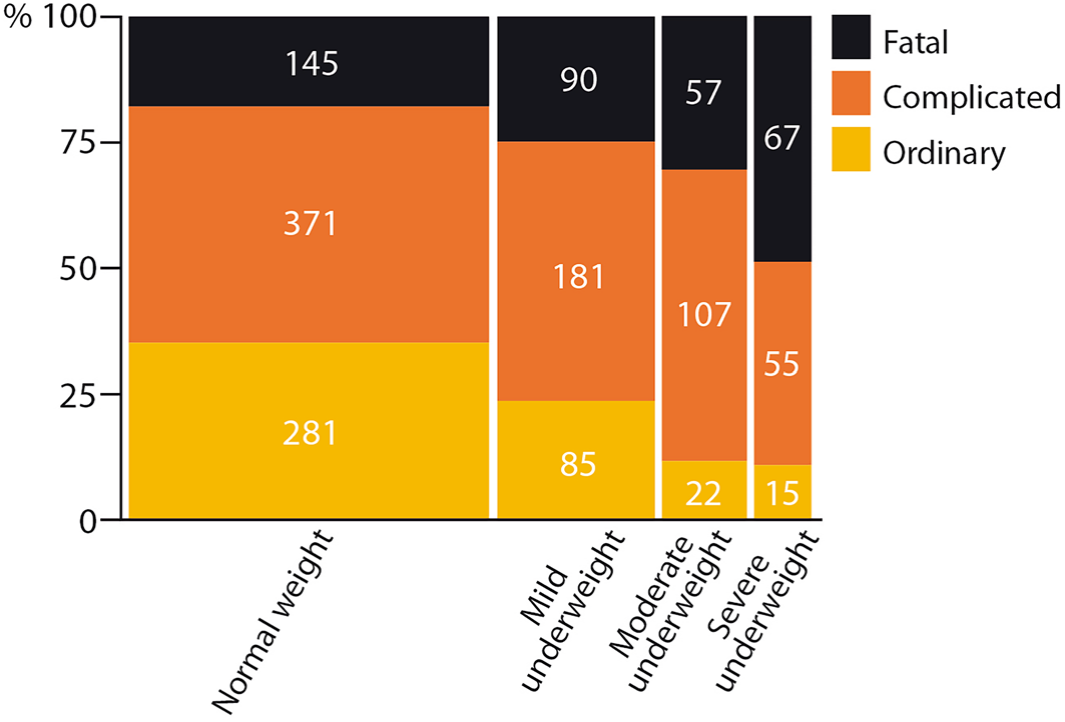
Clinical course according to weight-for-age category in all patients. *Ordinary*: Daily improvement and diminishing irritability. No seizures after day 3 of treatment. No focal neurological signs at any time. *Complicated*: Fever (axillary temperature > 37.4 °C), or irritability over 5 days. Seizures after day 3 of treatment, or focal seizures any time. Focal neurological signs at any time. Another focus of infection. Causal micro-organism resistant to the administered antibiotic. *Fatal*: Death caused by bacterial meningitis.

In all patients combined (Table 5), there was a decrease in the proportion of females, CSF glucose concentration, and blood hemoglobin concentration in accord with to the worsening category of underweight (*p* < 0.0001 for each). The change commenced in mild underweight, increased in moderate underweight, and reached its maximum in severe underweight. A similar stepwise change, albeit an increase, was seen for the presence of focal neurological signs (*p* = 0.03), another focus of infection (*p* < 0.0001), severe neurological sequelae (*p* = 0.002), any neurological sequelae (*p* < 0.0001), and deaths (*p* < 0.0001). A stepwise change along with worsening category of underweight was likewise observed in the change of the clinical course of disease (Fig. 2), from ordinary to complicated to fatal (*p* < 0.0001).

**Table 5:** Changes in variables associated with outcome from childhood bacterial meningitis according to weight-for-age category in all study patients.

	Normal weight	Mild underweight	Moderate underweight	Severe underweight	*p* value
Weight-for-age z-score	+ 1 to − 1	< − 1 to − 2	< − 2 to − 3	< − 3	
Number	808 (54)	356 (24)	189 (13)	137 (9)	
Female	391 (48)	163 (46)	66 (35)	42 (31)	< 0.0001
Ill before admission, days	3 (2–5)	4 (2.1–7)	5 (3–8)	5 (3–14)	< 0.0001
CSF^a^-leucocytes, /mm^3^	1800 (465–4758)	1750 (500–5140)	1430 (360–3263)	1200 (305–3800)	0.066
CSF-glucose, mg/dL	17.5 (7.0–41·0)	11.0 (5.4–25.8)	10.9 (5.6.22.3)	10.0 (5.8–24.8)	< 0.0001
CSF-protein, mg/dL	163 (100–245)	175 (98–288)	209 (135–280)	178 (122–263)	0.022
**Causative bacteria**	713 (54)	322 (24.5)	163 (12.5)	117 (9)	< 0.0001
*Haemophilus influenzae*	328 (46)	126 (39)	61 (37)	40 (34)	
*Streptococcus pneumoniae*	204 (28.5)	124 (38.5)	57 (35)	46 (39)	
*Neisseria meningitidis*	133 (18.5)	47 (14.5)	21 (13)	10 (9)	
Other bacteria	48 (7)	25 (8)	24 (15)	21 (18)	
B^b^-haemoglobin, g/dL	9.0 (7.5–11.0)	8.5 (7.0.10.2)	8.0 (6.2–9.4)	7.0 (6.0.8.5)	< 0.0001
B-leucocytes × 10^9^/L	14.6 (8.8–20.4)	15.2 (9.0–21.3)	15.0 (10.2–23.4)	14.3 (9.3–21.0)	0.44
B-glucose, mg/dL	93 (71–120)	90 (71–114)	81 (69–105)	81 (57–104)	< 0.0001
D-vitamin, mean	93 (80–119)	111 (89–141)	98 (71–115)	84 (51–113)	0.034
D-vitamin, all, nmol/L	95 (82–119)	115 (89–151)	111 (67–117)	84 (51–117)	0.019
HIV positive	··	11/146 (8)	8/102 (8)	12/63 (19)	0.006
Altered consciousness	586/784 (75)	263/350 (75)	141/183 (77)	106/136 (78)	0.82
Glasgow Coma Score	13 (10–15)	12 (8–15)	11 (7–15)	12 (8–14)	0.001
Seizures before or at admission	284/782 (36)	168/351 (48)	91/184 (49)	63/134 (47)	0.0001
Days of GCS^c^ < 15	2 (1–4)	2 (1–5)	2 (1–6)	3 (1–5)	0·0008
Seizures at ward	272/575 (53)	168/321 (52)	103/178 (58)	83/130 (64)	0·002
Focal neurological signs	125/776 (16)	62/349 (18)	35/182 (19)	36/135 (27)	0.030
Other focus of infection^d^	241/656 (37)	146/309 (47)	91/166 (55)	75/121 (62)	< 0.0001
Death	145/808 (18)	89/356 (25)	57/189 (30)	67/137 (49)	< 0.0001
Severe neurological sequelae	47/652 (7)	26/265 (10)	20/131 (15)	12/68 (18)	0.002
Deafness	47/558 (8)	21/225 (9)	12/118 (10)	7/58 (12)	0.78
Any neurological sequelae	191/648 (29)	98/263 (37)	60/129 (47)	37/68 (54)	< 0.0001
Any hearing loss	144/507 (28)	76/184 (41)	41/90 (46)	21/46 (46)	0.0002

Data are presented as medians (interquartile range), or number with the condition/all examined (percentage) and compared between the categories using contingency table or Kruskal–Wallis test, as appropriate.

^a^Cerebrospinal fluid. ^b^Blood. ^c^Glasgow Coma Score.^d^Pneumonia,or cellulitis, or otitis media, or impetigo.

## Discussion

Our main results depict an especially high prevalence of underweight in Angola and clearly show how in LatAm and Angola underweight was associated with a severe course of BM with more deaths, in LatAm already by the mild form of underweight. The difference in the relative importance of underweight for increasing deaths in LatAm and Angola derived from the presence of other, locally more predominant risks such as admission disease severity in Angola. In Finland, the BM patients´ WAZ-scores were overall within the expected in optimal growth^12–14^.

In Latin America, the 12.6% prevalence of a WAZ-score < − 2 identified moderate-severe underweight as an additional concern in need of medical attention in the BM patients. Although not directly comparable, the LatAm prevalence of 12.6% of moderate-severe underweight is similar to the 2019 regional estimate of a 7.1% prevalence of moderate-severe stunting (= height-for-age [HAZ] <  − 2, indicating chronic undernutrition) in under 5-year-olds by the joint report of UNICEF/WHO/World Bank Group^16^. The difference from 12.6 to 7.1% in the data collected in 1996–2003^5^ and in 2019^16^, respectively, could point to a small reduction in regional growth failure.

In Angola, the prevalence of any underweight (58.6%) was double that in Latin America (31.8%), and severalfold that in Finland (12.9%), or in “optimal growth” (15.8%, Table 1). Half of the underweight (in 31.3% of all patients) was moderate-severe. This is essentially the same figure as the 32% prevalence of moderate-severe stunting (HAZ < 2) both in an Angolan community study of stunting in under 2-year-olds^17^ and the 2019 regional estimates in under 5-year-olds for Central Africa^16^. In contrast to Latin America, in Angola the relatively frequent parasitic infections have probably contributed to poor growth, possibly via environmental enteric dysfunction^18,19^. Our findings suggesting some lowering in the prevalence of undernutrition in Latin America, together with the persisting level in Angola, agree with the report of a reduction in growth faltering in middle-income countries but less so in low-income countries^20^.

A recent systematic review and meta-analysis of sex differences in undernutrition found that boys were more likely to present this condition than girls, albeit with some regional differences^21^. The mechanisms behind the difference are unknown. Our data replicated the finding of lower WAZ-scores in boys in LatAm and Angola.

The significant association between a lower WAZ-score with a lower blood hemoglobin level in Finland and LatAm suggests poor nutrition as the source of anemia. The lack of the same association in Angola points to such causes of anemia which do not necessarily affect body weight, like endemic malaria, or sickle cell disease (SCD). In Angola the BM patients with SCD had a significantly lower median hemoglobin level than those without SCD (*p* < 0.0001), but their WAZ-scores did not differ (*p* = 0.74, data not shown).

Our study is among the few documenting clearly how underweight was associated with findings depicting a severe course of BM already on admission and throughout the hospital stay. These findings highlight the early and possibly irreversible negative effects of undernutrition in the fight against severe infections. Whether the damage could be moderated by supplementary feeding or access to optimal intensive care remains unknown^22^.

Undernutrition´s deleterious effects in increasing the susceptibility, severity and risk of death from infectious diseases are related with a weakened immune response^11^. In undernutrition, all the immune system´s functions ranging from being a gut-barrier, recognizing and eliminating of noxious pathogens, fighting infection and creating immune memory are debilitated^23^. Besides the lack of macronutrients, the suboptimal functioning is related with that of micronutrients and changes in the gut microbiota^23^. Several micronutrients, such as vitamin D, retinol, vitamin C, selenium and zinc are of special importance supporting both the adaptive and innate immune systems^24^. In adults, the Covid-19 epidemic has underlined “poor nutrition” as a risk for severe and fatal infection^23,24^.

The moderate-severe stages of growth failure (z-score < − 2) have understandably received the most attention, also in the guidelines for its management^25^. However, also the mild stage of underweight (WAZ < − 1 to − 2) merits attention because it significantly increased BM deaths in LatAm. In our combined data, the change for the worse in the variables predicting an adverse course of BM began already in mild underweight (Table 5 and Fig. 2). Clinically, mild underweight is an easily missed diagnosis, as demonstrated by the clinical impression of “any malnutrition” not being recognized before the WAZ-score was well into moderate underweight (− 2.53, SD 1.39). Importantly, even a clearly stunted child (HAZ-score < 2, indicating chronic undernutrition) can clinically appear only small and not especially thin^26,27^. This stresses the need for objective measurements for nutritional characterization, not only clinical judgement, even in areas where clinicians are used to dealing with undernourished children. If the harm from mild underweight is overlooked, these patients will be left without appropriate nutritional management.

The previous data of the numerical effect of growth failure on increasing deaths in BM is scarce and based on slightly different definitions. In Ethiopia^28^, severe wasting (= weight-for-height z-score [WHZ] <  − 3, indicating acute undernutrition) increased BM deaths 2.8 times (95% CI 1.1–7.7), which is similar to our odds in Angola of 1.92 (95% CI 1.21–3.04) for severe underweight (WAZ < − 3). In Peru, “malnutrition”, not defined in more detail, significantly (*p* = 0.03) increased deaths^29^. In comparison, our results of a 5.31-fold increase in deaths by severe underweight (WAZ < 3) in Latin America is clearly higher. A variable effect of growth failure for increasing BM deaths in different children is theoretically a possible explanation, which we cannot fully discard by our data. To us, a more likely explanation for the discrepancy are other important predictors of death at different locations, as shown in our multivariate analysis (Table 4). In Angola, where the patients arrived so ill that all deaths occurred after a median of 18.5 h of treatment^30^, a Glasgow Coma Score < 13 on admission was the dominant predictor, with the highest OR of 5.68, compared with a secondary role for severe underweight, with an OR of 2.11. In Latin America, severe underweight dominated, with an OR of 6.69, compared with a Glasgow Coma Score < 13, with an OR of 3.95.

A possible inaccuracy in our results could stem from having calculated the WAZ-scores based on admission weights, which after incipient BM with fever and lesser consumption of liquids at home may present some underestimation of the actual weight. In any case, the comparability of the WAZ results remains intact, as the same measure was used at all sites. Another shortcoming in our analysis was the lack of length/height measurements. This made it impossible to define the exact type of undernutrition as stunting (HAZ < − 2, chronic undernutrition) or wasting (WHZ < − 2, acute undernutrition). Oedema was not specifically registered, but cases of kwashiorkor were very infrequent at the time of the studies in both Latin America and Angola.

Since the child´s nutritional status is of paramount importance in general for normal development and well-being and influences the course and mortality of many diseases^20,26^, experts recommend nutritional screening for all hospitalized patients^31^. In children with severe diseases, also the risk of undernutrition should be evaluated. This requires methods which are better performed in hospital wards, such as STRONGkids^32^_,_ and a careful measurement of length/height. In contrast, even busy admission departments weigh all children, independently of the workload. The weight allows a quick (within seconds) determination of weight-for-age by Anthro programs, which serves as an alert for further, more detailed nutritional evaluation and an indication for appropriate management.

Based on our results, ending undernutrition could remove an important risk for poor outcome from BM, especially in resource-poor areas. Yet, the 2019 report by UNICEF/WHO/World Bank Group^17^ and the 2021 Lancet report on Maternal and Child Undernutrition Progress^20^ showed insufficient advancement towards the Goal to End Malnutrition by 2030. Now, with the Covid-19 pandemic having affected, as predicted, the upholding of vaccinations and food security^33–35^, the need to implement evidence-based interventions for improving maternal and child nutrition^19^ is increasingly urgent.

## Data Availability

The data used and analyzed during the current study are available from the corresponding author upon a reasonable request.
